# Pulsed Electric Field Enhanced Cross‐Linking of Alginate and Fish Gelatin for Anti‐Oxidative Activities and Entrapment of Nutrients

**DOI:** 10.1002/fsn3.71879

**Published:** 2026-05-13

**Authors:** Qiaoxia Zhan, Matt Jellecoe, Shan He, Liang Jia

**Affiliations:** ^1^ School of Food and Pharmacy Zhejiang Ocean University Zhoushan Zhejiang China; ^2^ College of Medicine and Public Health Flinders University Bedford Park South Australia Australia; ^3^ Faculty of Science, Technology, and Engineering Charles Darwin University Casuarina Northern Territory Australia

**Keywords:** anti‐oxidation, biopolymer, cross‐linking, nutrient entrapment, pulsed electric field

## Abstract

Pulsed electric field (PEF) processing has emerged as a promising non‐thermal technology capable of modifying biomolecular structures and enhancing functional properties in food systems. In this study, PEF was applied to fabricate cross‐linked alginate–fish gelatin materials, and their structural characteristics, antioxidative activity, and nutrient entrapment capacity were compared with those produced using conventional homogenization. Structural analysis revealed that PEF processing generated smaller particle sizes (~80 nm) compared with homogenization (250–550 nm) and produced more compact microstructures with lower surface area (4678.3 vs. 8230.2 m^2^/g) and porosity (0.0973 vs. 0.2366 cm^3^/g). These structural differences significantly improved the functional performance of the material. Nutrient entrapment capacity increased from 21% to 33% for minerals and from 15% to 24% for vitamins in PEF‐treated samples compared with homogenized samples. In addition, antioxidative activity measured by FRAP and ABTS assays showed a notable enhancement in PEF‐processed materials. The results demonstrate that PEF can effectively promote protein–polysaccharide interactions and improve the structural and functional properties of alginate–fish gelatin complexes. This study provides a novel non‐thermal strategy for developing protein–polysaccharide materials with enhanced nutrient delivery and antioxidative functionality for food applications.

## Graphical abstract

This study demonstrates a novel PEF‐based method to fabricate cross‐linked alginate‐fish gelatin materials with compact microstructure. Compared to homogenization, PEF processing significantly improved antioxidant activity and enhanced nutrient entrapment capacity for minerals (33% vs. 21%) and vitamins (24% vs. 15%).

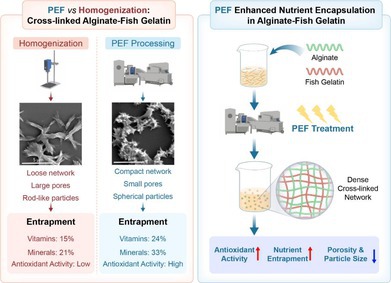

## 1 Introduction

The usage of Pulsed electric field (PEF) is one of the short duration non‐thermal processes that has been very popularized in commercial scenarios. This process comparatively has fewer effects in the food processing arena because of negligible effect on the nature of the processed food (Vashisht et al. 2024). As compared to the standardized thermal methodologies, this non‐thermal technique highlights certain advantages like biocompatibility, low energy usage and easy customization. Jafari et al. (2024) showcased the use of the non‐thermal PEF process to treat food products like sterilization of milk and fruit juices. Along the time, this novel technology has been extensively explored at both the commercial and experimental levels. The utilization of PEF equipment has been constantly increasing from testing phase to fully functional phase, especially in the industrial sectors. This step was initially introduced as a pre‐processing phase to manufacture bioactive ingredients. It was able to enhance the production of washed bioactive compounds to optimize the cell permeabilization (Brückner and Griehl 2023). Baltacıoğlu et al. (2024) studied the consequences of PEF pretreatment on fried yellow‐ and purple‐fleshed potatoes. Their study demonstrated that PEF treatment significantly reduced oil content by at least 20% and 24% in yellow fleshed and purple fleshed potatoes, while enhancing phenolic compounds.

Recent years have shown the effect of PEF treatment on various proteins, including wheat flour proteins (Achayuthakan et al. 2023), soy proteins (Belobrajdic et al. 2024), and legume proteins (Bravo‐Núñez and Gómez 2021). For example, Wang et al. (2023) showcased the combined PEF treatment at 10 kV/cm with pH adjustments significantly enhanced the solubility of commercial soy protein isolate, increasing it from 26.06% to 70.34%. One of the processes of PEF treatment shows that the ionized charge residues or repulsed electrostatic interactions can lead to secondary and tertiary morphological modifications in proteins. It has been stated that the protein physicochemical attributes are a function of the protein structure. Nevertheless, the impact of the PEF processing on physicochemical properties of protein‐polysaccharide interacting materials was rarely studied previously.

Fish gelatin has been widely used for nutrient encapsulation in functional foods due to its good biocompatibility and film‐forming ability. For example, Liu et al. (2021) developed fish gelatin/sodium alginate double‐network gels to encapsulate probiotics, improving their thermal stability and survival in simulated gastric fluids. Similarly Hadidi et al. (2021), encapsulated *Lactobacillus acidophilus* in alginate–fish gelatin beads, enhancing probiotic viability during heating and storage. The nutrient‐trapping ability of fish gelatin systems can be further improved through cross‐linking with polysaccharides. For instance, Peng et al. (2024) reported improved oxidative stability and loading capacity of fish oil using genipin‐crosslinked gelatin–polysaccharide complexes, achieving loading capacities up to 44.3% and encapsulation efficiencies of 90.9%. Likewise Oliver‐Cadena et al. (2024), developed fish gelatin films cross‐linked with transglutaminase and laminated with amylopectin, resulting in enhanced mechanical properties and water resistance for food packaging applications. Despite these advances, most reported studies rely on chemical cross‐linking agents or conventional mechanical processing methods, which may involve additional reagents, complex processing conditions, or limited control over microstructure formation. In contrast, PEF processing can induce structural modifications in biomolecules through electrostatic interactions, ion migration, and molecular rearrangements, potentially facilitating protein–polysaccharide self‐assembly without the need for chemical cross‐linkers.

Previous work has also explored vortex fluidic device (VFD) processing to promote interactions between biomolecules and fabricate functional biopolymer materials. For example, VFD processing has been used to enhance enzymatic hydrolysis and promote the formation of structured biopolymer systems through high shear stress and microfluidic mixing (Joseph 2021). These studies demonstrated that VFD can significantly influence molecular interactions and material morphology in food and biomaterial systems. However, VFD relies primarily on mechanical shear forces and thin‐film microfluidics to induce molecular interactions (Cao et al. 2021). In contrast, PEF processing operates through high‐voltage electric pulses that induce electroporation, dipole alignment, and electrostatic interactions between biomolecules, which may lead to different structural outcomes in protein–polysaccharide systems. Despite the promising capabilities of PEF processing, the application of PEF for constructing cross‐linked alginate–fish gelatin systems for nutrient encapsulation has not been previously reported.

Therefore, this study aims to investigate the feasibility of using PEF to fabricate cross‐linked alginate–fish gelatin materials and to evaluate their structural characteristics, antioxidative activity, and nutrient entrapment capacity compared with conventional homogenization treatment.

## 2 Materials and Methods

### 2.1 Materials

Nutrient supplements of Berocca, enriched with vitamins, and Kid's multi‐minerals, enriched with minerals, were contributed by Chemist Warehouse (Australia). Fish gelatin (food grade) was obtained from Woolworth supermarket (Australia). Alginate was acquired from Sappire Bioscience (NSW, Australia). Milli‐Q water was obtained via the interaction between protein and polysaccharides.

### 2.2 Fabrication of Cross‐Linked Alginate‐Fish Gelatin Materials

Fish gelatin and alginate were amalgamated with aqueous solutions. The ratio between the fish gelatin to alginate was 2:0.75 (w/w), having the combined final concentration of 0.18 g/mL. The first step of the fabrication process includes the pre‐mixing of 10 mL of 2% (w/v) fish gelatin solution and 6 mL of 0.125 (w/v) alginate solution by stirring for 15 min at room temperature. The PEF system designed by PulseMaster Pty. Ltd. (Hapert, The Netherlands) was used in this study. The experimental system included a control panel, a power source for high‐voltage pulse, an oscilloscope to determine the output, and a parallel plate chamber to treat the material. The distance between two electrodes was 2.5 cm to obtain uniform pulse discharges. Around 100 mL premixed solution was placed in the treatment chamber and treated with PEF at a 58% duty ratio for 6 min at 14 kV/cm. The PEF signal with an input frequency of 900 Hz and monopolar mode was able to generate a pulsed square waveform. The customized homogenization technique was employed to compare with VFD method. These experiments have also used the same amount for PEF, which was subsequently homogenized (homogenizer T25 digital ULTRA‐TURRAX) at 13,500 rpm for 600 s. The samples obtained from the PEF and homogenization processes were obtained, and then subsequently frozen and dried before their use.

### 2.3 Dynamic Light Scattering (DLS) Technique

The distribution of the obtained samples from the PEF and homogenization processes was done at 25°C using DLS (Nano ZS90, Malvern instruments, Worcester, UK). These samples were then treated with a He‐Ne 633 nm wavelength laser at a detector angle of 173°. The Malvern zeta sizer instrument was able to determine the scattering of the different particle sizes with respect to the time‐dependent fluctuations of light.

### 2.4 Scanning Electron Microscopy (SEM)

We characterized the output from the last step obtained in both liquid and freeze‐dried forms using optical images. An Inspect FEI F50 SEM (PS216) instrument was used to determine the SEM image under different magnifications. The spot size and the input voltage were 2.0 and 5.0 kV, respectively. For the observation of freeze‐dried microstructures, the samples were first freeze‐dried and then rapidly immersed in liquid nitrogen for approximately 2 min to facilitate fracture. The frozen samples were subsequently cut into pieces of approximately 5 × 5 × 2 mm (length × width × thickness) using a surgical blade. The fractured surfaces were mounted on SEM stubs and sputter‐coated with a thin platinum layer (~2 nm) prior to imaging to improve conductivity and image resolution.

### 2.5 Brunauer, Emmett, and Teller (BET) Analysis

The total surface area and porous nature of the frozen and dried samples were calculated using the BET analytical technique (TriStar II, Micrometric Ltd., Lincoln LN6 3RX, United Kingdom). The frozen and dried samples were then made into powder via liquid nitrogen. Then, 40 mg of the crushed sample was positioned at the end of a BET tube, with subsequent 12 h evacuation of the BET tube. Finally, the BET tube was placed in the analyzer having the adsorptive gas, bath temperature and equilibration interval as N_2_, 77 K and 20 s, respectively.

### 2.6 Fourier Transform Infrared Spectroscopy (FTIR)

FTIR spectra of frozen and dried materials were done via a spectrometer equipped at a room temperature of 25°C. The instrument was equipped with a diamond crystal for attenuated total reflection. The spectral range, scans, and resolution were fixed at 400**–**4000 cm^−1^, 40 and 4 cm^−1^, respectively. Then, around 10 mg of the prepared samples were placed on the diamond crystal for measurement purposes. The instrument having a background and a sample spectrum also had the same spectral range, scans, and resolution as that of the diamond crystal.

### 2.7 Viscosity Measurement

The viscosity of the cross‐linked alginate‐gelatin materials was analyzed by a Brookfield viscometer assembled with a thermo‐container and programmed temperature controller (LV‐DVIII, HT‐60, HT‐110FR, respectively; Brookfield Engineering Laboratories Inc.; Middleboro, MA, U.S.A). The thermo‐container was integrated with a sample chamber and a spindle (HT‐2 and SC4‐18, respectively; Brookfield Engineering Laboratories Inc.; Middleboro, MA, U.S.A). The measurement unit was cooled via a cooling plug assembly (HT‐26Y, Brookfield Engineering Laboratories Inc.; Middleboro, MA, U.S.A) attached to a pressurized air nozzle. The temperature for controlling the heating profile was set between 20°C and 40°C with an increment of 2°C/min. The sample weight was set at 2 g and the shear rate, and a gap were set to 20.00 rad/s and 4 mm, respectively.

### 2.8 Nutrients Entrapment by Cross‐Linked Alginate‐Fish Gelatin Materials

The multi‐mineral and vitamin nutrient supplement of Kid and Berocca, respectively, were crushed into powder before the entrapment studies. The samples were formed by mixing gelatin, tannic acid, and each ground nutrient supplement with water at fixed ratios. The ratio between gelatin to tannic acid to each nutrient supplement was 2:0.375:1.25 (w/w/w). The experimental step consisted of the pre‐mixing of 10 mL of 2% (w/v) gelatin solution and 3 mL of 0.125 (w/v) tannic acid solution, followed by subsequently mixing 1.25 g of powdered nutrient supplement. The samples were then poured onto a borosilicate glass tube (20 mm OD, 17 mm ID) in the VFD via the jet feeds. The borosilicate glass rotated at a speed and flow rate of 7000 rpm and 0.3 mL/min, respectively. The tube was operated at room temperature and fixed at a tilt angle of 45°. These experimental conditions were obtained by optimizing the processing conditions to develop the VFD‐assisted non‐covalent cross‐linked alginate–fish gelatin material. The experiments carried here had the same concentrations as that of the PEF processing, where the homogenization process (homogenizer T25 digital ULTRA‐TURRAX) had the same conditions as that of the aforementioned homogenization‐produced cross‐linked tannic acid–gelatin material (13,500 rpm for 10 min at 25°C). The developed liquid samples obtained from the PEF and homogenization processes were collected and then frozen and dried.

### 2.9 Quantification of Entrapped Nutrients in Cross‐Linked Alginate‐Fish Gelatin Materials

The quantification of entrapped nutrients in cross‐linked alginate–fish gelatin materials was carried out using scanning electron microscopy equipped with energy‐dispersive X‐ray spectroscopy (SEM‐EDS) (Inspect FEI F50, Bruker Cooperation, Billerica, MA, USA). SEM‐EDS was selected because it enables simultaneous visualization of the microstructure and elemental mapping of the sample, allowing the detection and semi‐quantitative analysis of mineral elements associated with the encapsulated nutrients. This technique has been widely used to evaluate the spatial distribution and relative abundance of elements in food and biomaterial matrices.

In the present study, the elemental signals detected by EDS (e.g., P, Si, Mg, and Zn for mineral supplements) were used as indicators of the entrapped nutrients within the alginate–fish gelatin matrix. The results were expressed as atomic percentage (at%), representing the relative proportion of each detected element in the analyzed region. By comparing the elemental percentages between samples produced via PEF and homogenization treatments, the relative nutrient entrapment capacity of the different materials could be evaluated.

### 2.10 Measurement of Antioxidative Activity

The antioxidative activity of the samples was evaluated using the ferric‐reducing antioxidant power (FRAP) assay and the ABTS radical scavenging assay. These two methods were selected because they represent complementary mechanisms for assessing antioxidant capacity: FRAP evaluates the reducing power of antioxidants by measuring their ability to convert ferric ions (Fe^3+^) to ferrous ions (Fe^2+^), while the ABTS assay measures the ability of antioxidants to scavenge free radicals. The combined use of these assays provides a more comprehensive assessment of antioxidative properties in food biopolymer systems.

The FRAP assay was conducted according to Amamcharla and Metzger (2014) with minor modifications. The FRAP reagent consisted of 10 mM TPTZ dissolved in 40 mM hydrochloric acid, 20 mM ferric chloride, and acetate buffer (0.3 M, pH 3.6). The processed materials were mixed with the reagent at a ratio of 1:1:10 (v/v). Each test sample consisted of 1 mL of sample solution and 5 mL of FRAP reagent. The mixture was incubated at 37°C for 20 min, and the absorbance was measured at 593 nm using a spectrophotometer. The antioxidant capacity was calculated using a standard curve generated from ferrous sulfate solutions (100–1400 μM), and the results were expressed as FRAP values (μM Fe^2+^).

The ABTS radical scavenging activity was determined following the method of Dechakhamphu et al. (2023) with minor modifications. Briefly, the ABTS radical solution was prepared by mixing a 7 mM aqueous ABTS solution with 2.45 mM potassium persulfate (K_2_S_2_O_8_) and incubating the mixture in the dark at 25°C for 16 h. The resulting solution was diluted with ethanol to obtain an absorbance of 0.70 (±0.02) at 734 nm and equilibrated at 30°C. Subsequently, 0.3 mL of each sample was mixed with 1.2 mL of the ABTS solution, incubated for 6 min, and the absorbance was measured at 734 nm. The ABTS radical scavenging activity was calculated according to the following equation:

$$\text{ABTS radical scavenging ability}\;\left(\%\right)=\left[\frac{A_{\text{control}}-B_{\text{sample}}}{A_{\text{control}}}\right]\times 100\%$$

where *A* is the absorbance of the blank sample and *B* is the absorbance of the testing samples.

### 2.11 Data Analysis

Triplication was applied for detection purposes where the template of mean with standard deviation was determined to present data and subjected to the least significant difference (LSD) and one‐way variance analysis (ANOVA) using v15 MINITAB Statistical Software. The F value at probability (*p* < 0.05) was utilized to calculate the statistical significance.

## 3 Results and Discussion

### 3.1 Synthesis and Physical Attributes of Cross‐Linked Alginate‐Fish Gelatin

**FIGURE 1:**
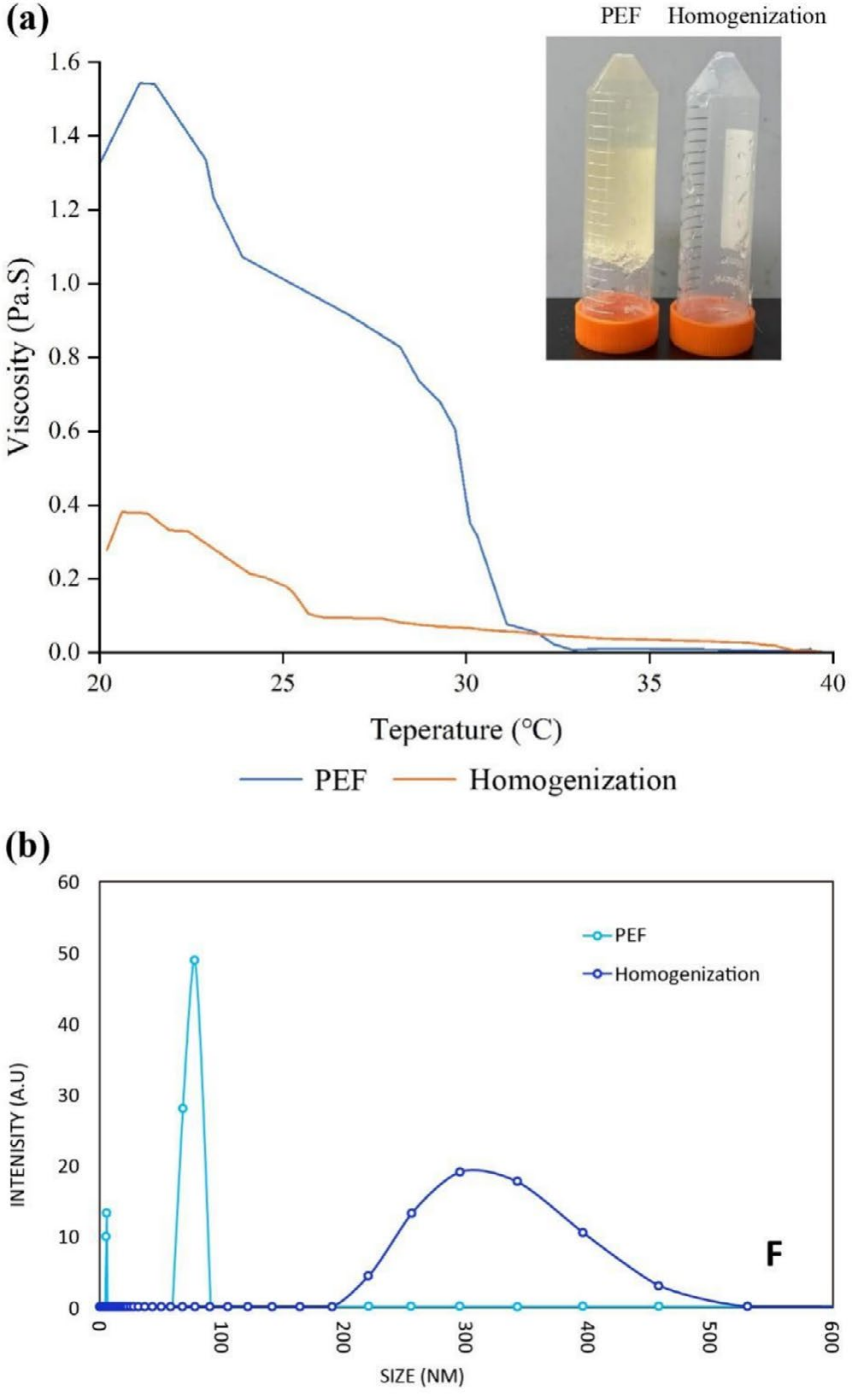
Physical properties of cross‐linked alginate–fish gelatin systems obtained by PEF treatment and homogenization. (a) Viscosity data and representative visual appearance of the samples after processing; the photograph was taken 12 h after preparation. (b) Dynamic light scattering (DLS) analysis.

The interaction between polysaccharides and proteins has been widely explored to enhance the functional properties of food materials. For example, protein–polysaccharide composite systems have been applied in biodegradable food packaging films and hydrogel matrices for environmental remediation (Roy et al. 2024; Wang et al. 2022). Cross‐linking between fish gelatin and alginate has also been reported in previous studies, demonstrating improved gel stability and mechanical properties (Basu et al. 2022). However, most fabrication approaches rely on conventional mechanical mixing or chemical cross‐linking, and alternative processing strategies for controlling the microstructure of these materials remain limited. In the present study, the PEF‐treated samples exhibited a visibly more viscous and gel‐like structure compared with the materials produced by homogenization. When the containers were inverted, the PEF‐processed material remained attached to the bottom of the container, indicating the formation of a stable gel network, whereas the homogenized sample flowed downward due to its lower viscosity (Figure 1a). These observations suggest that PEF processing promotes stronger interactions between alginate and fish gelatin. Such differences in rheological behavior may be attributed to enhanced cross‐linking and molecular interactions induced by the electric field during PEF processing. High‐intensity electric fields can alter protein conformation, promote exposure of reactive functional groups, and enhance electrostatic interactions between proteins and polysaccharides (Taha et al. 2022; Wang et al. 2023). Similar improvements in viscosity and gel stability resulting from increased cross‐linking density have been reported in other biopolymer systems (Muguda et al. 2021; Vinner and Malik 2018). These mechanisms likely contribute to the formation of the more stable gel structure observed in the PEF‐treated alginate–fish gelatin materials.

The above differences in viscosity of cross‐linked alginate‐fish gelatin compound, amid using PEF process and homogenization was kept after 12 h at 25°C. The solutions developed freshly achieved significant variability in viscosity (Figure 1a). The temperature ranging from 20°C to 30°C assisted in obtaining the viscosity of cross‐linked alginate‐fish gelatin compound developed via PEF process was much more than that developed via homogenization process. The viscosity of the cross‐linked alginate–fish gelatin materials was measured using a Brookfield viscometer under controlled temperature conditions, as described in Section 2.7. As shown in Figure 1a, the viscosity values were obtained from the steady‐state measurements recorded at different temperatures during the heating profile (20°C–40°C) at a shear rate of 20 rad s^−1^. For example, at 25°C, the viscosity of the PEF‐treated sample was approximately 1.1 Pa s, whereas the homogenization‐treated sample exhibited a significantly lower viscosity of approximately 0.2 Pa s, indicating the formation of a stronger gel network in the PEF‐processed material.

**FIGURE 2:**
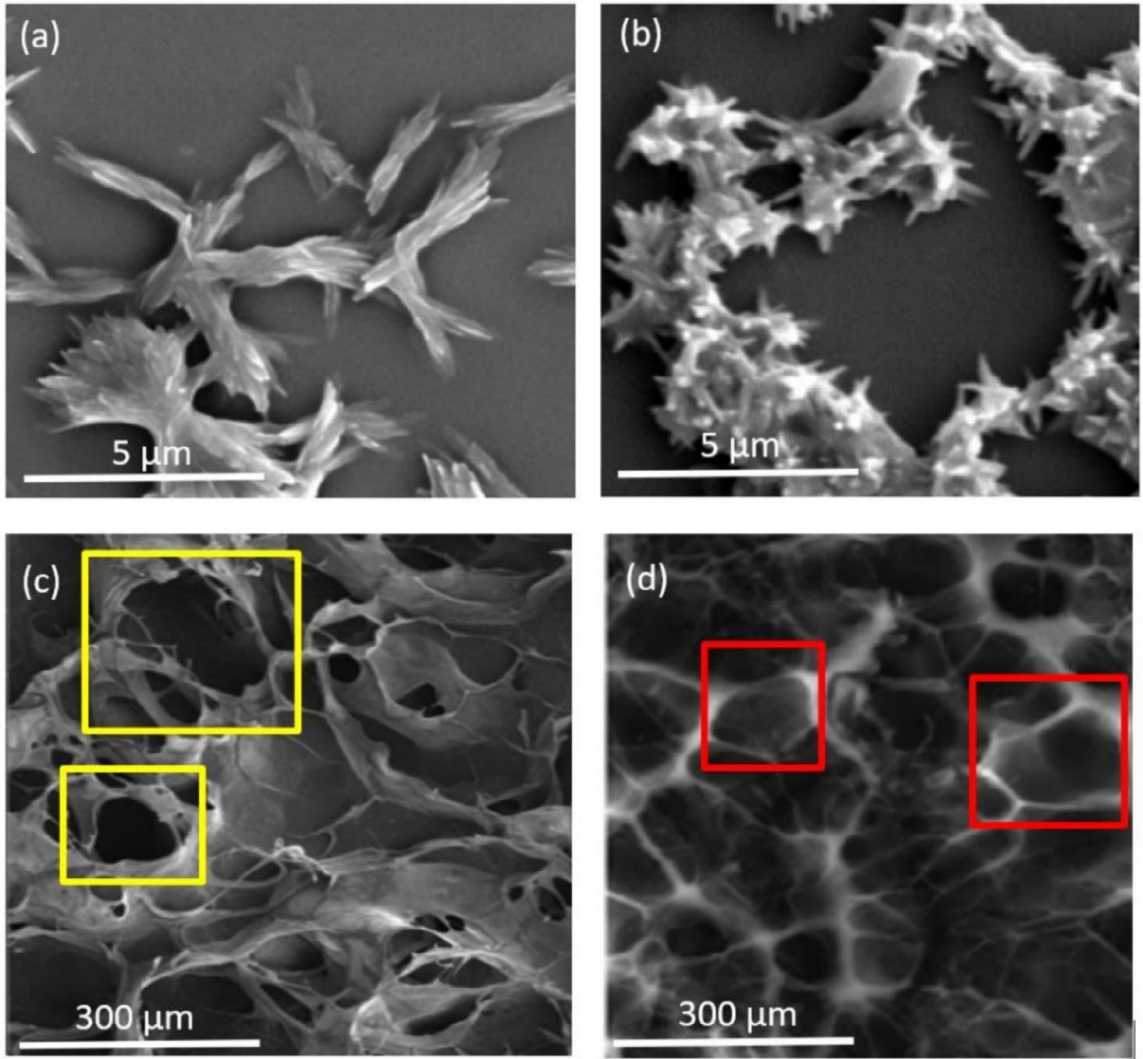
SEM images of cross‐linked alginate–fish gelatin structures. All samples were prepared by drop‐casting onto a silicon wafer, dried (overnight or freeze‐dried), and sputter‐coated with platinum prior to SEM analysis. (a) Homogenization, oven‐dried. (b) PEF treatment, oven‐dried. (c) Homogenization, freeze‐dried. (d) PEF treatment, freeze‐dried.

Studies have indicated that increased cross‐linking contributes to greater viscosity and enhanced stickiness in cross‐linked biopolymers (Muguda et al. 2021). A higher degree of cross‐linking, achieved by utilizing elevated concentrations of alginate in high internal phase emulsions made from modified starch, results in noticeable increases in both viscosity and shear modulus as the alginate concentration rises from 0% to 2% (w/w) (Vinner and Malik 2018). The elevated amount of alginate also resulted in the formation of high internal phase emulsion after the method. The alteration in the liquid from dripping down to solid gel attached to the end of the container was like the present study (Figure 1a). Akin output was showcased using alginate in developing hybrid supramolecular nanofibers (Kupferberg et al. 2024). It is seen from the output given in Figure 2a that an enhanced crosslinking capability of alginate advocated on fish gelatin while they were exploited for PEF, as compared with the homogenization process.

The particle dimensions of cross‐linked alginate‐fish gelatin produced through the PEF and homogenization process were analyzed via DLS (Figure 1b), revealing a significant difference of approximately 80 nm for PEF and 250–550 nm for homogenization. This underscores the ability of PEF to achieve significantly smaller particle sizes.

### 3.2 Scanning Electron Microscopy (SEM)

The structural dimension of cross‐linked alginate‐fish gelatin was analyzed using SEM, as shown in Figure 2. SEM images captured immediately after production on different processing platforms reveal distinct structures: homogenization resulted in aggregated rod‐like formations (Figure 2a), while the PEF produced enclosed, spicular‐shaped structures (Figure 2b). PEF processing achieves the formation of enclosed, spicular‐shaped structures due to its unique mechanism of action. PEF involves the application of short bursts of high‐voltage electric fields to the material, which can induce significant physical and structural changes of polysaccharides, such as starch, at the molecular and microscopic levels (Maniglia et al. 2021). The high‐intensity electric fields in PEF cause electroporation, where biopolymers develop temporary pores. This can lead to the redistribution of molecules, promoting the reorganization of biopolymeric structures such as alginate and gelatin into specific patterns. In addition, PEF can influence cross‐linking by promoting ion migration and interaction, particularly in systems like alginate, which relies on ionic cross‐linking (e.g., calcium ions). This also encourages the development of compact, enclosed structures (Abourehab et al. 2022). We note that PEF technology has been shown to significantly influence protein–saccharide interactions, enhancing functional properties in food systems. Previous studies have demonstrated that PEF treatment can induce several specific structural modifications in proteins and protein–polysaccharide systems. High‐intensity electric fields can partially unfold protein molecules by disrupting weak non‐covalent interactions such as hydrogen bonds and electrostatic interactions, leading to the exposure of buried hydrophobic residues and reactive functional groups (Taha et al. 2022). Such structural rearrangements can alter the secondary structure of proteins, typically decreasing α‐helix content while increasing β‐sheet or random coil structures (Wu et al. 2016). The exposure of these functional groups enhances intermolecular interactions, including hydrophobic interactions, electrostatic attraction, and potential covalent cross‐linking between proteins and polysaccharides. In alginate‐based systems, PEF has also been reported to facilitate ion migration and promote stronger ionic cross‐linking between negatively charged alginate chains and positively charged protein domains, resulting in more compact and stable biopolymer networks. These combined structural effects contribute to the formation of smaller particles and more organized microstructures, as reflected in the DLS (Figure 1b) and SEM (Figure 2) results observed in the present study.

**FIGURE 3:**
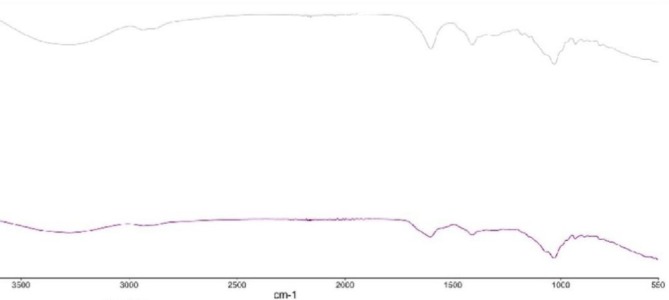
FTIR spectra of alginate–fish gelatin interacting systems obtained by PEF treatment (top spectrum) and homogenization (bottom spectrum).

The total area was frozen and dried through homogenization technique, with the entire area being irregular with substantial porosity/surface area ca 31,300 m^2^ (Figure 2c), pores highlighted in yellow squares. Comparatively, the total area of the freeze‐dried prototypes formed through PEF had smoother and regular surface with lower porosity/surface area of ca 2600 m^2^ (Figure 2d), pores highlighted in red squares. The prototypes depicted lucid structural difference prior to (Figure 2a,b) and subsequent to the freeze‐drying process (Figure 2c,d). PEF processing facilitates a more regular microstructure in protein‐polysaccharide interactions due to its ability to control molecular interactions and alignments without causing extensive disruption. PEF generates strong electric fields that influence charged molecules like proteins and polysaccharides. These fields can induce dipole alignment and help molecules orient themselves in a more ordered arrangement. Proteins often carry surface charges, and polysaccharides (like alginate) are usually polyanionic. PEF enhances their interactions by aligning charges, promoting consistent binding and reducing random aggregation (Zhou and Pang 2018). Furthermore, The electric pulses can generate microfluidic flows at interfaces, enhancing the mixing and interaction of proteins and polysaccharides. This dynamic interaction prevents phase separation and promotes uniform microstructural assembly (Xu et al. 2023). By leveraging these mechanisms, PEF provides precise control over molecular interactions, enabling the formation of regular and well‐defined microstructures in protein‐polysaccharide systems. This makes it particularly advantageous for applications requiring consistent textural and functional properties. It is seen from Figure 2b that these regular, spicular and circular structures, as compared to the rods with variant sizes and open‐structured samples via homogenization process shown in Figure 2a. The similarity of the shred cross‐linked materials obtained after the processing are depicted in Figure 2c,d, are similar to the ones shown in Figure 2a,c, respectively. The output with homogenous, spicular morphology subsequent to the PEF processing (Figure 2b) caused the reassembling of the defined morphology with small porosities. The width of thin, clear and even outlines shown in Figure 2d agreed with the diameter of the cross‐linked and encapsulated elements in Figure 2b. Although, the prototypes with different anomalous shapes of rods and open structure subsequent to the homogenization technique (Figure 2a) consists of enlarged pores, without lucid and even boundaries between them (Figure 2c). As the Szerlauth et al. (2023) had stated on the processed material during PEF processing results in various physical attributes, with rigorous, absolute and uniform shredding, any kind of chemical interaction (formation of bonds) in the middle of the components, as for deploying homogenization process. This interaction was observed through FT‐IR analysis of the alginate–fish gelatin composites (Figure 3). Characteristic gelatin bands corresponding to amide I (~1650 cm^−1^) and amide II (~1540 cm^−1^) were detected, while alginate exhibited typical carboxylate stretching bands around ~1600 cm^−1^ and ~1410 cm^−1^. In the composite samples, slight shifts and intensity changes of these peaks were observed, indicating electrostatic interactions between the carboxyl groups of alginate and the amino groups of gelatin. Additionally, variations in the broad O—H/N—H stretching region (~3200–3400 cm^−1^) suggest the formation of hydrogen bonding between the two biopolymers. These spectral changes confirm the molecular interaction between gelatin and alginate in the cross‐linked materials.

The entire area and porosity of these elements subsequent to the freeze‐drying process were quantified by the BET characterization. The entire sector of the prototypes exploited by the homogenization (8230.2 m^2^/g) was around twice that of the prototypes exploited via PEF (4678.3 m^2^/g). Similar behavior was observed for the porosity, where the prototypes processed via the homogenization technique at 0.2366 cm^3^/g was more than the one developed via PEF, 0.09732 cm^3^/g.

It is also informative to compare the present PEF approach with vortex fluidic device (VFD) processing previously reported for biomolecular systems (Joseph 2021). VFD processing generates intense shear stress and thin‐film microfluidic environments that enhance mixing and molecular interactions. In contrast, PEF processing induces structural modification through electric‐field‐driven mechanisms such as electroporation, dipole orientation, and electrostatic interactions between charged biomolecules. These distinct mechanisms can lead to different microstructural outcomes. In the present study, PEF processing produced more compact structures with reduced porosity and enhanced nutrient entrapment compared with conventional homogenization, suggesting that electro‐physical interactions induced by PEF may provide an alternative pathway for constructing protein–polysaccharide networks.

From a process engineering perspective, PEF also offers advantages in terms of scalability, as PEF systems are already widely used in industrial food processing for continuous treatment of liquids and semi‐liquid materials (Taha et al. 2022). In contrast, VFD processing typically relies on laboratory‐scale rotating tube systems, which may require more complex engineering modifications for large‐scale production. Therefore, the use of PEF may provide a more practical route for the scalable fabrication of protein–polysaccharide cross‐linked materials in food and biomaterial applications.

### 3.3 Anti‐Oxidative Activity of Alginate‐Fish Gelatin Interacted Materials

**FIGURE 4:**
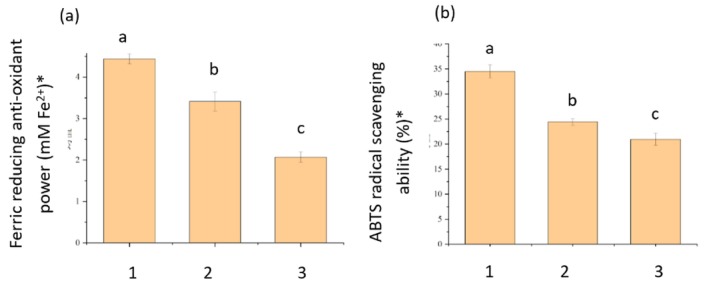
Antioxidant activity of alginate–fish protein interacting systems, determined by ferric reducing antioxidant power (FRAP) (a) and ABTS radical scavenging activity. (1) Alginate–fish protein interacting system produced by pulsed electric field (PEF). (2) Alginate–fish protein interacting system produced by homogenization. (3) Alginate alone (negative control). *Data are presented as mean ± standard deviation (*n* = 3). Different letters within each graph indicate significant differences among samples according to one‐way ANOVA followed by the LSD test (*p* < 0.05). Letters are not compared between graphs.

The antioxidative activity of alginate–fish gelatin interacting materials produced using PEF and homogenization treatments was evaluated using the FRAP and ABTS assays (Figure 4). The results showed that both composite materials exhibited higher antioxidative activity than alginate alone, indicating that the interaction between alginate and fish gelatin enhanced the antioxidant capacity of the system.

Furthermore, the alginate–fish gelatin materials produced using PEF treatment exhibited significantly higher antioxidative activity compared with those produced using homogenization (*p* < 0.05). In the FRAP assay, the PEF‐treated sample showed the highest ferric‐reducing antioxidant power, while the homogenization‐treated sample demonstrated moderate activity, and alginate alone exhibited the lowest activity. A similar trend was observed in the ABTS radical scavenging assay, confirming that the PEF treatment enhanced the antioxidant performance of the composite materials.

The improved antioxidative activity observed after PEF processing may be attributed to structural modifications induced by the electric field. PEF treatment can promote partial unfolding of protein structures and exposure of reactive amino acid residues, which can enhance radical scavenging capacity (Taha et al. 2022; Wu et al. 2016). Additionally, PEF can facilitate stronger interactions between proteins and polysaccharides by enhancing electrostatic interactions and hydrogen bonding, thereby stabilizing antioxidant‐active functional groups within the composite structure (Zhou and Pang 2018). Previous studies have also reported that interactions between alginate and proteins can enhance antioxidative properties due to synergistic effects between functional groups present in both biomolecules. Alginate contains abundant carboxyl and hydroxyl groups, whereas proteins provide multiple functional groups including amino, carboxyl, sulfhydryl, and aromatic residues originating from amino acid side chains. These functional groups can participate in radical scavenging and metal‐chelating reactions, and their cooperative interactions may enhance the overall antioxidative activity of the alginate–gelatin composite (Zhao et al. 2020; Gupta et al. 2020).

Overall, these results demonstrate that PEF processing is more effective than conventional homogenization in enhancing the antioxidative activity of alginate–fish gelatin interacting materials, likely due to structural modifications and enhanced molecular interactions induced by PEF (Taha et al. 2022).

### 3.4 Nutrients Entrapment Capacity of Cross‐Linked Alginate‐Fish Gelatin

Table 1 depicts the appearances of different cross‐linked alginate‐fish gelatin molecules with the trapped minerals and vitamins with the frozen and drying process. Darker colors are used to detect the trapped nutrients. The ground nutrient supplements of the minerals and vitamins exploited by PEF had a darker color as compared to the ones exploited by the homogenization technique. This showed the drastic increase in the nutrient entrapment ability of the interacted alginate‐fish gelatin molecule when they operated by PEF as compared to the operation by the homogenization process.

**TABLE 1:** Display of ground nutrient supplements of minerals and vitamins trapped via various interacting alginate–fish gelatin materials after freeze‐drying by PEF and homogenization.

	Interacted alginate‐fish gelatin	
	Homogenization treatment	PEF treatment
In the presence of minerals	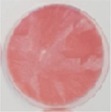	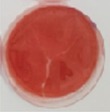
In the presence of vitamins	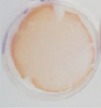	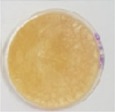

The nutrient trapping was characterized using SEM technique equipped with energy‐dispersive X‐ray spectroscopy. The quantified output (Tables 2 and 3) had a high similarity with the display of the prototypes in Table 1. The prominent changes in the color of the mineral trapped samples in the middle of those formed through a PEF and those formed through homogenization processing (Table 1) are shown via significant variation in the sum of the atomic weight percentages in Table 1 (21% for the homogenization sample, 33% for the PEF sample), with 6%, 3%, 1%, and 3% increase of P (13% to 7%), Si (9% to 6%), Mg (4% to 3%), and Zn (8% to 5%), respectively. Numerous processes have been highlighted to enhance the trapping ability of minerals in the gel systems that involve highly critical impregnation (Mao et al. 2020). The PEF methodology shown in Figure 4 and Table 1 demonstrated one more technique of this kind to improve the trapping ability of minerals. As compared to the minerals, the change in color in the vitamin trapped samples in the middle of the ones formed using a PEF and the ones formed using homogenization process was very less, as shown in Table 3. The sum of the atomic percentage changes is minor at 9%, with 3% and 4% enhancement of Vitamin H and B1 (11% to 8%), Vitamin B6 (6% to 4%), and Vitamin B13 (7% to 3%), respectively. As a result of the morphology of Vitamin B6 (with P), Vitamin H and B1 (with S), and Vitamin B13 (with Co), thus a popular process to utilize the percentages of cobalt (Co), sulfur (S), and phosphorus (P) to showcase the percentages of Vitamin B13, Vitamin H and B1, Vitamin B6, respectively, to calculate with SEM integrated with energy‐dispersive X‐ray spectroscopy (Redan et al. 2024).

**TABLE 2:** The percentage of trapped element in interacted alginate–fish gelatin prepared using homogenization and a PEF.

	Homogenization	PEF
P: Phosporous (percentage of testing material)	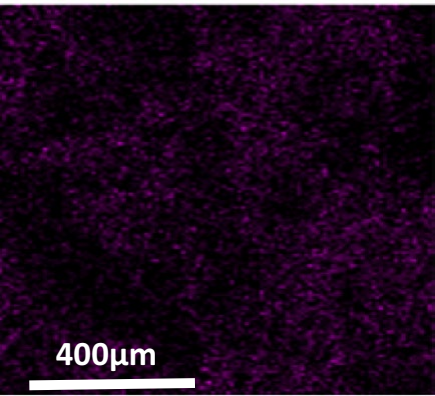 (7%)	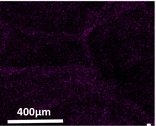 (13%)
Si: Silicon (percentage of testing material)	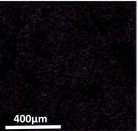 (6%)	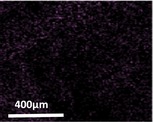 (9%)
Mg: Magnesium (percentage of testing material)	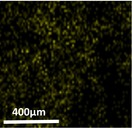 (3%)	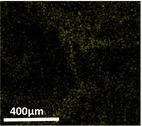 (4%)
Zn: Zinc (percentage of testing material)	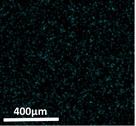 (5%)	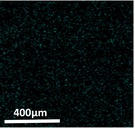 (8%)
Sum percentage	21%	33%

**TABLE 3:** The percentage of each trapped vitamin in interacted alginate‐fish gelatin prepared using homogenization and PEF.

	Homogenization	PEF
Vitamin H and B1, represented by S: Sulfur (percentage of testing material)	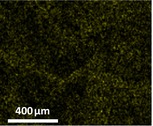 (8%)	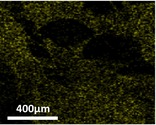 (11%)
Vitamin B6, represented by P: Phosphorous (percentage of testing material)	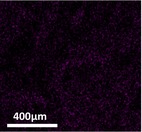 (4%)	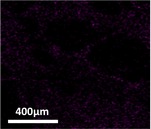 (6%)
Vitamin B13, represented by Co: Cobalt (percentage of testing material)	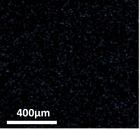 (3%)	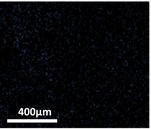 (7%)
Sum percentage	15%	24%

## 4 Conclusion

The usage of the PEF technique of cross‐linked alginate–fish gelatin has been developed to trap nutrients and compare with utilizing standardized homogenization techniques. Observed by SEM, cross‐linking with a closed circle was established in the samples using PEF, and incomplete cross‐linking with an open rod structure was observed in the samples developed via homogenization process. The SEM images also showed with the total surface area of the pores and their porosity produced using PEF processing were both substantially reduced compared with prototypes developed through homogenization technique. The anti‐oxidative activity and trapping ability of nutrients for minerals and vitamins for cross‐linked alginate–fish gelatin material developed via PEF were both prominently enhanced than the ones developed through homogenization process. Overall, the output determined that PEF techniques laid out a novel order for interaction between polysaccharide and protein with the potential for food processing.

## Author Contributions

**Liang Jia:** software, visualization. **Qiaoxia Zhan:** conceptualization, methodology, investigation, formal analysis, writing – original draft. **Shan He:** supervision, funding acquisition, project administration, writing – review and editing. **Matt Jellecoe:** resources, data curation, validation.

## Funding

This work was supported by the National Key Research and Development Program of China (Grant No. 2024YFD2101204).

## Conflicts of Interest

The authors declare no conflicts of interest.

## Data Availability

The data that support the findings of this study are available from the corresponding author upon reasonable request.
